# Factors linked to suicide risk in a diverse sample of psychiatric patients

**DOI:** 10.1192/j.eurpsy.2023.702

**Published:** 2023-07-19

**Authors:** M. Pontiggia, S. Bonaldi, C. Gramaglia, P. Zeppegno, F. Madeddu, R. Calati, S. Magliocca

**Affiliations:** ^1^Department of Psychology, University of Milan-Bicocca, Milan; ^2^ Institute of Psychiatry, Università del Piemonte Orientale; ^3^S.C. Psichiatria, Azienda Ospedaliero Universitaria Maggiore della Carità, Novara, Italy; ^4^Department of Adult Psychiatry, Nîmes University Hospital, Nîmes, France

## Abstract

**Introduction:**

Suicidal behavior is a global public health problem. Among the most investigated theories for the explanation of suicide there is the interpersonal-psychological theory of suicide (IPTS) by Thomas E. Joiner. IPTS focuses on 3 variables related to lethal suicide attempt: thwarted belongingness, perceived burdensomeness and acquired capability for suicide.

**Objectives:**

We aimed to understand which variables were mostly related to suicidal ideation (SI) and suicide attempts (SA) in a sample of psychiatric patients.

**Methods:**

A sample of 80 psychiatric patients including inpatients (n=18), outpatients (n=21) and patients from various Italian rehabilitative psychiatric communities (n=41) was recruited between June 7^th^, 2021 and September 12^th^, 2022. We administered a battery of various scales, including State-Trait Anxiety Inventory, STAI, Beck Depression Inventory, BDI, Reasons For Living Inventory, RFLI, Acquired Capability for Suicide Scale-Fearlessness About Death, ACSS-FAD, Rosenberg self-esteem scale, RSES, Mental pain questionnaire, MPQ, Interpersonal Needs Questionnaire, INQ. For each scale we compared a) patients with and without lifetime SI; b) patients with and without history of SA. Then, we performed two logistic regression models (stepwise backward method), one for SI and one for SA.

**Results:**

Both patients with SI and SA have higher anxiety (STAI), depression (BDI), mental pain (MPQ), perceived burdensomeness (INQ), thwarted belongingness (INQ), fearlessness about death (ACSS_FAD) and lower self-esteem (RSES), beliefs about coping strategies (RFLI) and moral objections (RFLI). Depression (BDI) and beliefs about coping strategies (RFLI) were the variables most strongly associated with SI; higher fearlessness about death (ACSS-FAD) and low self-esteem levels (RSES) were the variables most strongly associated with history of SA. The SI model was able to predict 84% of ideation cases; the SA model was able to predict 74% of the attempts.

**Conclusions:**

The results supported that improving the ability to cope with suicidal thoughts is a key component of therapeutic work with suicidal patients. In addition, according to the IPTS, the history of SA has been particularly explained by fearlessness about death. Hence it may be important to focus on these aspects in suicide prevention.

**Disclosure of Interest:**

None Declared

